# Reduction of venous pressure during the resection of liver metastases compromises enteric blood flow: IGFBP-1 as a novel biomarker of intestinal barrier injury

**DOI:** 10.6061/clinics/2017(10)10

**Published:** 2017-10

**Authors:** Hermes Vieira Barbeiro, Marcel Autran César Machado, Heraldo Possolo de Souza, Fabiano Pinheiro da Silva, Marcel Cerqueira César Machado

**Affiliations:** I Departamento de Emergencias Clinicas, Faculdade de Medicina FMUSP, Universidade de Sao Paulo, Sao Paulo, SP, BR; II Hospital Sirio Libanes, Sao Paulo, SP, BR

**Keywords:** Liver, Inflammation, Intestinal Barrier, Bacterial Translocation, IGFBP-1

## Abstract

**OBJECTIVES:**

Disruption of the intestinal barrier and bacterial translocation commonly occur when intestinal blood flow is compromised. The aim of this study was to determine whether liver resection induces intestinal damage.

**METHODS:**

We investigated intestinal fatty-acid binding protein and insulin-like growth factor binding protein levels in the plasma of patients who underwent liver resection.

**RESULTS:**

We show that liver resection is associated with significant intestinal barrier injury, even if the Pringle maneuver is not performed.

**CONCLUSION:**

We propose the use of insulin-like growth factor binding protein-1 as a novel biomarker of intestinal damage in such situations.

## INTRODUCTION

Several reports have revealed that in several clinical situations, such as burns 1,2, hemorrhagic shock 3,4, acute pancreatitis 5,6, aortic dissection and aortic surgery 7, sepsis 8 and after a Pringle maneuver during liver resection surgery 9, the intestinal barrier is disrupted when intestinal blood flow is compromised.

Because blood transfusion secondary to intraoperative blood loss is associated with early- and long-term complications in patients subjected to liver resections, several strategies, such as total 10 or intermittent 9 Pringle maneuvers and the reduction of central venous pressure, have been used to minimize blood loss. During the Pringle maneuver, however, reduction of intestinal perfusion results in intestinal barrier dysfunction and endotoxemia 9. Similarly, we hypothesized that reduction of central venous pressure during liver resection followed by reduction of arterial pressure would also reduce intestinal blood flow and damage the intestinal barrier. The aim of this study was to investigate whether lowering of the central venous pressure is sufficient to promote acute intestinal barrier dysfunction in patients subjected to liver resection without the Pringle maneuver.

## PATIENTS AND METHODS

Eighteen patients who underwent liver surgery at Hospital Sirio Libanes in São Paulo (Brazil) were eligible for inclusion in this study. All patients had colon cancer. Surgeries were performed to resect one or multiple liver metastases. Only three patients, who required the Pringle maneuver, were excluded. Perioperative profile of the study population is shown in Table 1. Informal consent was obtained from every patient. The protocols employed followed the Hospital Sirio Libanes guidelines, were approved by their ethics committee (protocol number 2013-54) and were performed in accordance with the principles of the National Council of Animal Experiment Control (Concea).

Central venous pressure was maintained at a low level (below 10 mmHg) during liver resection to reduce bleeding and to facilitate liver tissue manipulation. Seventeen patients underwent major liver resection (>3 segments), and one patient underwent a minor resection (segmentectomy). During the procedure, catheters were inserted to monitor the central venous and radial arterial pressures. Parenchymal resection was performed using bipolar cautery, sutures and vascular staples. Blood samples were obtained from the arterial line at the start of the procedure and 8h later. Blood was collected in EDTA-containing vacuum tubes, kept on ice, and centrifuged at 4°C; the plasma was stored at -70°C. Intestinal fatty acid binding protein (IFABP), a specific marker of intestinal barrier dysfunction 8, was measured by an enzyme-linked immunosorbent assay (ELISA kit, MyBIOSource, USA), according to instructions provided in the manual. Insulin-like growth factor binding protein (IGFBP) plasma measurements were performed using Milliplex technology (Merck, Genese Diagnostics, Darmstadt, Germany). Plasma IL-6 levels were determined by an enzyme-linked immunosorbent assay (R&D Biosystems, USA).

### Statistical analysis

Data are expressed as the means±SD. The results were compared using Student’s t-test. A *p* value of <0.05 was considered statistically significant. Statistical analyses were performed using GraphPad Prism 5.0 for Windows.

## RESULTS AND DISCUSSION

**Figure 1:**
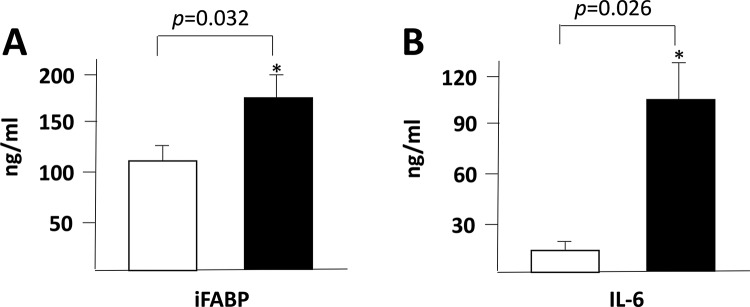
Intestinal fatty-acid binding protein (IFABP, Figure 1A) and IL-6 (Figure 1B) plasma levels at the beginning of liver resection (in white) and 8 h later (in black).

**Figure 2:**
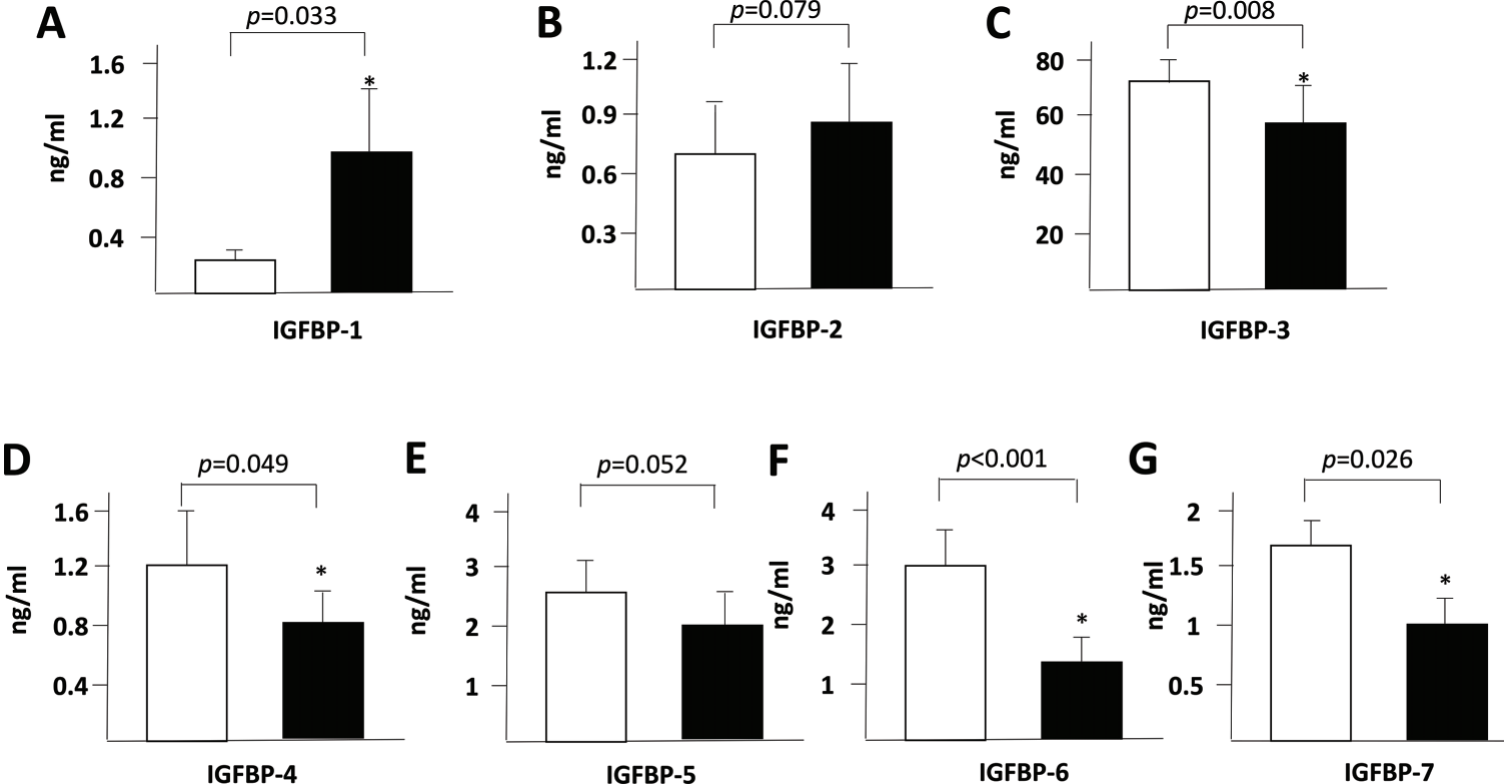
Insulin-like growth factors binding proteins (IGFBP) 1 to 7 plasma levels (Figures A to G) at the start of liver resections (in white) and 8 hours later (in black).

We found a significant increase in the plasma levels of IFABP (Figure 1A), IL-6 (Figure 1B) and IGFBP-1 (Figure 2A) at 8h after the start of the surgeries. In contrast, the IGFBP-3, IGFBP-4, IGFBP-6 and IGFBP-7 plasma levels were decreased after the surgical procedure (Figure 2 C, D, F and G).

Previous studies have shown that, in patients undergoing liver resection, total or intermittent Pringle maneuvers are associated with an increase in IFABP plasma levels due to the loss of enterocyte membrane integrity. In addition to the Pringle maneuver, alternative strategies have been used during hepatic resection to minimize intraoperative blood loss, such as the pharmacological reduction of central venous pressure. However, we hypothesized that this strategy would reduce the arterial blood pressure, which might also compromise the intestinal blood flow. As previously demonstrated, reduction of intestinal blood flow is followed by intestinal barrier dysfunction and bacterial translocation 11, and several reports have shown that IFABP is an accurate biomarker of intestinal damage 8,12.

In the present study, we observed a significant increase in IFABP blood levels, at 8h after hepatic resection, with a reduction of the central venous pressure during liver transection even without the Pringle maneuver (Figure 1A), and a similar trend was observed for the IL-6 plasma levels. These results indicate that liver resection induces a significant local and systemic inflammatory response, even affecting organs located at sites distant from the surgical procedure, such as the intestines.

IGFBPs are important physiological regulators of the interaction of insulin-like growth factors (IGFs) with their receptors within the gastrointestinal tract and liver 13,14, and thus, we decided to investigate them as novel potential biomarkers of acute intestinal dysfunction. The seven cloned mammalian IGFBPs are implicated in cell proliferation and survival as well as in several other cellular responses. IGFBPs are secreted by human intestinal epithelial cells and may leak into the systemic circulation in situations involving intestinal damage. In the present study, we observed a significant increase in IGFBP-1 blood levels after liver resection (Figure 2A). However, other IGFBPs presented a postoperative blood level decrease after hepatic resection (Figure 2).

In a previous study, intestinal epithelial cell damage and endotoxemia were demonstrated to occur after the Pringle maneuver during liver resection 9, but patients submitted to liver resection without the Pringle maneuver did not present intestinal damage. Those patients, however, were more hemodynamically stable and did not require the Pringle maneuver. In our study, all patients were operated on without the Pringle maneuver, independent of their hemodynamic conditions. In our patients, central venous pressure was maintained below 10 cm H2O by pharmacological manipulation, and intestinal barrier dysfunction was detected. Elevation of IGFBP-1 has also been observed during critical illnesses 15, most likely due to systemic inflammation. However, in our study, the IGFBP-1 levels had returned to normal (data not shown) at 24h after liver resection. Elevations of IGFBP-1 after burn injury 16 may also (at least in part) be related to acute intestinal dysfunction 17.

In conclusion, the present study demonstrated that, even without the Pringle maneuver, intestinal barrier dysfunction most likely occurs secondary to the reduction of intestinal blood flow induced by pharmacological manipulation of the central venous and arterial pressure.

In the modern era of liver surgery, the Pringle maneuver has been restricted to situations in which major bleeding occurs. Future investigations are necessary to establish whether intestinal barrier dysfunction plays a role in the etiology of infectious or noninfectious perioperative complications. Intestinal barrier dysfunction may be an important concern in patients with small liver remnants. In fact, endotoxin translocation, secondary to intestinal barrier dysfunction, may induce liver damage 18 and further jeopardize liver function. An alternative approach would be to perform a selective intrahepatic Glissonian approach 19 and proceed to liver resection without a central venous pressure reduction, thereby maintaining the intestinal perfusion and epithelial cell integrity. However, this approach still needs to be further investigated.

A previous study in patients with biliary malignancy demonstrated that bacterial translocation can predict the occurrence of postoperative infectious complications after liver resection 20.

Thus, we propose that novel techniques to perform liver resections without the Pringle maneuver and without reduction of the central venous pressure should be explored because both of the latter procedures compromise the intestinal integrity. IFAPB is a reliable marker of intestinal injury even before such injury can be detected by histological analysis. The gut is the main producer of IGFBP-1, although other organs can produce it in smaller amounts. We suggest that IGFBP-1 is a novel marker of intestinal damage, but further studies are necessary to confirm this finding.

## AUTHOR CONTRIBUTIONS

Barbeiro HV performed the experiments. The remaining authors designed the project and wrote the manuscript.

## Figures and Tables

**Table 1:** Perioperative profile of the study population (means). Standard deviations are listed in parenthesis.

**Age**	60.6 (29-77)
**Gender male/female**	7/9
**Blood loss (mL)**	515 (100-1500)
**Number of resected segments**	3 (1-4)
**Postoperative aspartate aminotransferase IU/L**	503 (76-1306)
**Postoperative alanine aminotransferase IU/L**	366 (59-697)
**Postoperative gamma-glutamyl transpeptidase IU/L**	144 (28-405)
**Postoperative total bilirubin mg/dL**	1.97 (0.22-4.93)
**Postoperative creatinine mg/dL**	1.51 (0.65-3.30)
